# Feasibility and acceptability of Narrative Exposure Therapy to treat individuals with PTSD who are homeless or vulnerably housed: a pilot randomized controlled trial

**DOI:** 10.1186/s40814-022-01043-x

**Published:** 2022-04-15

**Authors:** Nicole E. Edgar, Alexandria Bennett, Nicole Santos Dunn, Sarah E. MacLean, Simon Hatcher

**Affiliations:** 1 Clinical Epidemiology Program, Ottawa Hospital Research Institute, The Ottawa Hospital Riverside Campus, 1919 Riverside Drive, Suite 406, Ottawa, ON K1H 7W9 Canada; 2 School of Epidemiology and Public Health, Faculty of Medicine, University of Ottawa, Ottawa, ON Canada; 3 Ontario Institute for Studies in Education, University of Toronto, Toronto, ON Canada; 4 School of Journalism and Communication, Carleton University, Ottawa, ON Canada; 5 Department of Psychiatry, University of Ottawa, Ottawa, ON Canada

**Keywords:** Narrative Exposure Therapy, Complex post-traumatic stress disorder, Homelessness, Trauma-informed care

## Abstract

**Background:**

Diagnosed PTSD rates in people who are homeless are more than double that of the general population, ranging between 21 and 53%. Complex PTSD (cPTSD) also appears to be more common than PTSD. One treatment option is Narrative Exposure Therapy (NET), a brief trauma-focused psychotherapy which attempts to place trauma within a narrative of the person’s life. Our primary aim was to assess the feasibility and acceptability of recruiting people to a randomized controlled trial (RCT) of NET alone compared to NET augmented by a genealogical assessment. We hypothesized that incorporating a genealogical assessment may be more effective than NET alone in a population with predominately complex PTSD.

**Methods:**

This pilot RCT enrolled participants who were 18 years of age or older, currently homeless or vulnerably housed, and with active symptoms of PTSD. Participants were randomized to NET alone or NET plus a genealogical assessment. Rates of referral, consent, and retention were examined as part of feasibility. Demographic and clinical data were collected at baseline. Symptoms of PTSD, drug use, and housing status were re-assessed at follow-up visits. We conducted a thematic analysis of qualitative interviews of service providers involved in the study which explored barriers and facilitators of study participation.

**Results:**

Twenty-two potential participants were referred to the study, with 15 consenting to participate. Of these, one was a screen failure and 14 were randomized equally to the treatment arms. One randomized participant was withdrawn for safety. Attrition occurred primarily prior to starting therapy. Once therapy began, retention was high with 80% of participants completing all six sessions. Seven participants completed all follow-up sessions. Service providers identified a clear need for the treatment and emphasized the importance of trauma-informed care, a desire to know more about NET, and more communication about the process of referral.

**Conclusion:**

Recruiting participants who were vulnerably housed to an RCT of a trauma-based therapy was possible. Once therapy had started, participants were likely to stay engaged. We will incorporate the results of this trial into a conceptual model which we will test in a factorial study as part of the optimization phase of MOST.

**Trial registration:**

ClinicalTrials.govNCT03781297. Registered: December 19, 2018

**Supplementary Information:**

The online version contains supplementary material available at 10.1186/s40814-022-01043-x.

## Key messages regarding feasibility

- *What uncertainties existed regarding the feasibility?*
  Narrative Exposure Therapy (NET) is an individual trauma-focused psychotherapy recommended in guidelines for the treatment of post-traumatic stress disorder (PTSD). However, in people who are homeless or vulnerably housed, there have been no randomized controlled trials of trauma-focused therapies for PTSD. We wanted to find out if it was feasible to recruit and retain people who were homeless with PTSD in a randomized controlled trial of a trauma-informed therapy. We wanted to test the acceptability and feasibility of offering NET alone compared to NET plus a genealogical assessment. We also wanted to see if it was acceptable and practical to incorporate a genealogical assessment as part of NET.
- *What are the key feasibility findings?*
  The key feasibility finding is that it is possible to recruit and retain people who are homeless into a randomized controlled trial of a trauma-informed therapy. However, feasibility could be improved by a better process for engaging potential participants between referral and enrollment as about a third of the referred population were lost at this stage. Not having trained therapists available also delayed recruitment.
- *What are the implications of the feasibility findings for the design of the main study?*
  We will explicitly develop materials for use at the referral step by potential referrers. This will include an online training video which will address issues of trust and how to address them when discussing the potential study. We will also develop a process for training and recruiting therapists. Since this study was completed, we have done two further training workshops to create a pool of potential therapists in Ottawa. We will also engage with another site in Ontario to widen the population base of potential participants.

## Introduction

### Background

Homelessness is a rapidly growing problem in Canada with at least 235,000 individuals experiencing homelessness every year [1, 2]. In reality, this number is likely 3 to 4 times higher to account for individuals with no real prospect of permanent housing options or “hidden homelessness” [1]. In Canada, point-in-time counts have repeatedly shown year-over-year increases in homelessness [2]. Exacerbated by a nationwide housing crisis and a global pandemic, accompanied by a reduction in shelter beds and social services, 2021 reports have shown that the situation is continuing to worsen [3–5].

Individuals experiencing homelessness are more likely to have poor overall physical and mental health, increased mortality rates and to experience more barriers to accessing healthcare [6, 7] compared to their housed counterparts. In one study, nearly 50% of respondents indicated having 3 or more physical health conditions, and 52% indicated having a mental health diagnosis [6, 8]. Individuals who are homeless are more likely to be exposed to infectious diseases, such as tuberculosis or hepatitis A [6]; less likely to seek early treatment and less likely to receive care equivalent to those who are housed [9–12]; and face additional challenges with adherence to treatment regimens [13].

### Trauma in the homeless

Exposure to trauma is a nearly universal experience among the vulnerably housed. It is estimated that as many as 91% of individuals who are homeless have experienced at least one traumatic event [14] and up to 99% have experienced childhood trauma [15, 16]. Diagnosed post-traumatic stress disorder (PTSD) rates in the homeless are significantly higher than in the Canadian population, ranging between 21 and 53% [17–20], compared with a lifetime prevalence of 9.2% [21] in the general population. In addition to trauma before becoming homeless, the experience of being homeless increases the risk of exposure to traumatic events [22]. Many who are homeless are exposed to violence, with previous work showing 40% of individuals reporting being assaulted and 21% of women reporting being raped in the previous year [6]. Lastly, the experience of being homeless is itself traumatic due to the loss of shelter, safety, stability, and, often, social supports. Thus, being homeless continues to re-traumatize and victimize the individual [23].

In ICD-11, a distinction is made between PTSD and complex PTSD (cPTSD). cPTSD is characterized by experiencing trauma that is prolonged or repetitive from which escape is difficult or impossible (for example, repeated childhood sexual or physical abuse) [24]. This results in the symptoms of PTSD plus problems in affect regulation, negative self-beliefs, and difficulty sustaining relationships. In the homeless population, cPTSD appears to be more common than PTSD, with one survey of 206 homeless adults finding 60% diagnosed with cPTSD and 16% with PTSD [25, 26].

This subsequently impacts how individuals engage in healthcare services, with individuals often experiencing distrust of both people, including healthcare providers, and services [27]. It may also lead to self-medication with street drugs to address the symptoms of PTSD. There are also systemic barriers to accessing care which include difficulties finding transportation to appointments, institutional rules that effectively ban people who are homeless, feelings of stigmatization [9, 11], having proof of health insurance, and access to no-cost mental health services [28]. Most recently, the shift to primarily virtual mental healthcare has further isolated this population from accessing services. Providing therapy in this population should consider the challenges of structural exclusion that this population faces with respect to healthcare services and providers, as well as the unique symptoms associated with cPTSD.

### Narrative Exposure Therapy

Narrative Exposure Therapy (NET) is a brief trauma-focused psychotherapy and was developed based on principles derived from exposure therapy, cognitive behavior therapy, and testimony therapy [29]. NET attempts to place the trauma within a narrative of the person’s life. This therapy has been evaluated in traumatized populations with a focus on survivors of conflict and organized violence [29]. NET is recommended for the treatment of PTSD in several guidelines, such as the American Psychological Association guidelines [30] and the National Institute for Health and Care Excellence guidelines [31]. There are three therapeutic components which consist of education about the effects of trauma, constructing a biography, and narration of traumatic events. The autobiography is recorded by the therapist and is built upon with each subsequent reading. A focus of the therapy is to integrate the generally fragmented reports of traumatic experience into a coherent narrative and to bring about the habituation of emotional responses to reminders of the traumatic event [29]. There have been no trials of NET in homeless adults, although one study of NET with 32 street-involved children found a reduction in self-reported offences [32]. Anecdotal evidence of using this approach in the homeless population suggests that constructing an autobiography helps to give meaning to problems and provides the initial steps in constructing a core sense of belonging and identity. There is also some evidence that NET may have advantages in treating complex traumatization seen in disadvantaged populations compared to typical first-line therapy, such as Prolonged Exposure Therapy [33].

### Incorporation of genealogy

Genealogy has been used in family therapy [34] and counseling [35] to promote identity [36] and develop connections to ancestors. This can improve relationships with living relatives and potentially address the negative self-beliefs which are part of cPTSD. A systematic review of the acceptability of health and social interventions for people who were homeless found that having a positive self-identity improved links to services [27]. The link to those who have gone before is a common theme in indigenous health [37]. Previous experience of using problem solving therapy, with a focus on a sense of belonging, in Māori in New Zealand who had presented to hospital with intentional self-harm resulted in improved outcomes after a year compared to usual care. The focus on the sense of belonging helped to re-frame individuals’ narrative beyond the immediate family. The metaphor used by participants was that knowing about previous generations helped to deepen their roots so they were less likely to be blown over by life’s storms [38]. We want to test the idea that NET augmented by a genealogical assessment may be more effective than NET alone in the population of the vulnerably housed given that they suffer almost exclusively from complex PTSD.

### This trial

As of November 2021, there were no randomized controlled trials of trauma-focused therapies in people who are homeless with PTSD. The aim of this study is to test the feasibility and acceptability of referring and retaining to a study delivering community-based NET to individuals with PTSD who were homeless or vulnerably housed. We also extended the option of genealogist support to evaluate the potential impact of this experience on the development of their narrative and self-identity. This trial forms part of the preparation phase of a multi-phase optimization strategy (MOST) [39] for developing and delivering treatment for PTSD in people who are homeless using trauma-informed care. MOST differs from conventional intervention research in that there is an explicit phase prior to doing a randomized controlled trial which aims to create a conceptual model of the intervention which guides selection of which intervention components to examine, known as the preparatory phase [39, 40]. These intervention components are tested in a factorial trial, the optimization phase, to decide which are the most important. Finally, this optimized intervention is tested in a definitive randomized controlled trial (RCT). Using the outcomes from this study, a review of the literature on treating PTSD in people who are vulnerably housed [41], and consultation with people with lived experience, we will construct a model of the potential factors that may influence outcomes in this population when treating PTSD. We will then test this model to identify the most important factors in a factorial RCT. Following this, we will aim to complete a definitive trial of an optimized version of NET compared to optimized NET augmented by a genealogical assessment.

## Methods

### Trial design

We conducted a single-center feasibility, open-label pilot RCT with two parallel arms in Ottawa, Canada. The two arms were NET alone (NET) or NET plus a genealogical assessment (NET+G). This trial is reported using the Consolidated Standards of Reporting Trials extension for Feasibility and Pilot Trials (CONSORT) [42]. An RCT design was used to reduce potential selection bias for the augmentation of NET by a genealogical assessment.

### Participants

Participants were individuals with PTSD, diagnosed or suspected, who were experiencing homelessness or were vulnerably housed at the time of enrolment. Participants were referred to the study by clinical staff through Ottawa Inner City Health and the Royal Ottawa Hospital’s Psychiatric Outreach Team. Ottawa Inner City Health provides comprehensive health services, including mental health care to Ottawa’s homeless community. The Psychiatric Outreach Team is a community-based short-term service providing support and referrals to individuals who are homeless or vulnerably housed experiencing severe and persistent mental illness. Eligibility criteria are described in Table 1.

**Table 1:** Participant eligibility criteria

Inclusion criteria • 18 years of age or older • Be referred to the study by the Psychiatric Outreach Team or by Ottawa Inner City Health • Meet DSM-5 criteria for PTSD, as measured by the Mini-International Neuropsychiatric Interview (MINI) • Be homeless or vulnerably housed at the time of their screening visit, as measured by the Housing Status Questionnaire Exclusion criteria • Unable to speak and understand English • Unwilling to attend the Narrative Therapy session for a period of 6 weeks • Unwilling to return to a designated therapy location to complete study follow-up appointments • Presents to their study screening visit acutely intoxicated • Be, in the opinion of the investigator, unlikely to commit to a 12-week study • Poses a risk of harm to study staff or other clients	

### Interventions

Participants were randomized to one of two groups: Narrative Exposure Therapy only (NET) or Narrative Exposure Therapy augmented by a genealogist assessment (NET+G). Two therapists, a psychiatrist [SH] and a social worker [MSW – KB], who had received specialist training in NET, provided the therapy in the study. NET was delivered as described by Schauer et al. [29]. The components of NET as delivered in this study are outlined in Table 2. Participants attended weekly visits with the therapist at the location of their choosing. Flexibility in timing was offered to participants who found it challenging to attend weekly visits. Participants randomized to the NET+G arm were connected to a genealogist [MG, https://grandmasgenes.com/] after their baseline visit to complete a family history interview and voluntary genetic testing. Participants were provided with a full family history report detailing any information discovered through the interview and matching with public genealogy databases. For this study, Family Tree DNA [43] was used for genetic matching.

**Table 2:** Components of Narrative Exposure Therapy

Part 1—session 1	Completion of structured diagnostic measures (CAPS-5, PCL-5 with LEC and Criterion A) Psychoeducation and outline of the therapeutic plan
Part 2—session 2	Construction of the Lifeline—a visual biographical overview of significant life events
Part 3—sessions 3–6	*Sessions 3–5* Narration of the lifeline from birth through each event • Traumatic events are confronted and reprocessed until arousal response decreases • At follow-up sessions, the draft narrative is read through collaboratively, focusing on important events • The narrative is updated and corrected, providing more clarity with each read-through • This is repeated until a final version of the narrative is completed (by session 6) *Session 6* • The final narrative is read through entirely and signed by the participant and therapist • The participant is provided a copy of their narrative

Adapted from Schauer et al. [29]

Participants were met in the community at a mutually agreed upon location. Visit locations included shelters, outreach offices, community day programs, and our research office. Locations were chosen to minimize participant burden and increase feelings of comfort and safety. Any required travel costs were covered by the study.

After referral to the study, participants met with a trained research assistant to complete the informed consent process and screening procedures. Screening procedures to confirm eligibility involved a structured interview completed by the research assistant to confirm active symptoms of PTSD and housing status. Once eligibility was confirmed, participants completed assessments collecting demographics and evaluating general mental health, alcohol and substance use, quality of life, healthcare utilization, and cognitive state. We collected sociodemographic information on gender (male, female, transgender, non-binary), self-identified ethnicity, highest level of education completed, marital status, and medical history.

### Outcomes

The primary outcomes of this study were the feasibility and acceptability of completing an RCT of NET with people who were currently experiencing homelessness. The primary measure of feasibility was recruiting the planned sample size over 6 months, including potential participants identified and number of participants consented. In addition, we wanted to determine the acceptability of NET in this population, which we defined as at least 50% of those approached about the study consenting to take part. Lastly, we wanted to see if it was feasible to collect outcome data in this population, which we defined as restricting study drop-outs or lost to follow-ups to 25% of participants.

We also wanted to see if NET treatment in this population resulted in improved health-related outcomes compared to baseline measures and whether augmentation with a genealogical assessment provided further benefits than NET alone. Health-related outcomes included severity of PTSD symptoms, change in housing status, overall health, health-related quality of life, and rates of alcohol/drug misuse. Other secondary outcomes included creating a training manual for NET in this population that also included the incorporation of a genealogist.

Measures and timing of administration are outlined in Table 3. Housing status was collected prior to entry into the study and at each follow-up visit. Participants self-identified their housing among the following options: living in a shelter, living with a friend, living with family, supportive/transitional housing, paying for a space, no housing options, or other. Participants could select more than one option if applicable. The Addiction Severity Index interview [44] was completed at baseline as well as at weeks 4, 8, and 12 to evaluate both lifetime substance use and use in the past 30 days. We calculated the total number of days of use in the past 30 days for each time point to evaluate any changes in drug or alcohol use between visits. The SF-20 was used from February 2019 until September 2019, when it was replaced with the EQ-5D-5L. The decision was made to change measures as participants reported substantial difficulty in completing the SF-20 and interpreting the questions. The EQ-5D-5L is much shorter, with only 5 questions plus the visual analogue scale, and was much better tolerated as a measure. Participants who started with the SF-20 continued to use it through their follow-up time points.

**Table 3:** Time and events schedule

Visit/measure	Measure description	Screening/baseline	Week 1	Week 4	Week 8 follow-up	Week 12 follow-up
Housing status	A brief self-reported measuring describing participant’s housing status	X	-	X	X	X
MINI–PTSD Module	A structured interview assessing symptoms of PTSD in the past 30 days	X	-	-	-	-
CAPS-5	A structured interview, administered during therapy, to assess a traumatic event(s) and associated symptoms in the past 30 days	-	X	-	-	-
PCL-5 with Life Events Checklist (LEC)	A 20-item self-report evaluating the severity of PTSD symptoms over the previous 30 days; LEC assesses exposure to 16 events known to potentially result in PTSD	-	X	-	X *(PCL-5 only)*	X *(PCL-5 only)*
Demographics	Self-reported sociodemographic variables including gender, sexual orientation, ethnicity, education, and marital status	X	-	-	-	-
Addiction Severity Index	A semi-structured interview used to assess the severity of substance abuse problems, both lifetime and past 30 days	X	-	X	X	X
AUDIT	A brief 10-item self-report questionnaire assessing alcohol misuse	X	-	X	X	X
ADHD Self-Report Scale	An 18-item self-report questionnaire that assesses the inattentive (6 items) and hyperactive/impulsive (12 items) dimensions of ADHD	X	-	-	-	-
Montreal Cognitive Assessment	A brief interview assessing multiple cognitive domains including visuo-constructional skills, naming, memory, attention, sentence repetition, verbal fluency, abstraction, delayed recall, and orientation	X	-	-	-	-
Short Form Health Survey (SF-20) *or* EQ-5D-5L	The SF-20 was used from February 2019 to September 2019. This is a 20-item questionnaire that assesses various health outcomes, and the extent to which health-related problems interfere with daily life. The EQ-5D-5L was used from September 2019 to the end of the study. The EQ-5D-5L is a 5-item questionnaire that assesses health-related quality of life	X	-	X	X	X
Health Care Costs	A self-report questionnaire asking about missed time from work/volunteer/school and utilization of healthcare services	X	-	X	X	X

### Sample size

We planned to enroll 12 participants in each arm for a total sample of 24 participants. The sample size was decided on using Julious’ “rule of thumb” of 12 participants per arm in a pilot trial [45]. The study was not powered to test efficacy outcomes.

### Randomization and blinding

Randomization was completed by the Ottawa Methods Centre at the Ottawa Hospital Research Institute (OHRI) with allocations kept in sequential sealed opaque envelopes at the OHRI study office. Participants were randomized in a 1:1 allocation with no restrictions. After providing consent and confirming eligibility, participants were randomized by a trained research assistant according to the allocation in the sealed envelope.

### Statistical methods

All statistical analyses were conducted using IBM SPSS 26. Non-parametric data were described using frequencies and percentages. Continuous data were described using measures of central tendency (mean and standard deviation). Paired samples *t*-tests were conducted to explore any within-subject relationships for PCL-5 score and substance use patterns. To address the small sample size, bivariate relationships were detected using two-sided Fisher’s exact test (2 × 2 contingency tables).

### Qualitative evaluation

Qualitative interviews were planned with participants who completed the study and service providers including shelter, outreach, and day program staff. However, only interviews with service providers were completed due to issues with access created by the COVID-19 pandemic. The service provider interview evaluated their participation in the study, their perceived need for community-based NET to be implemented in this population, and what supports might be necessary to implement either a large-scale trial or a permanent service offering NET to individuals who are homeless or vulnerably housed. All interviews were completed and transcribed by a research assistant at arm’s length from the study (BW).

Thematic analytical techniques were used to identify, analyze, and report themes observed within the qualitative interview data [46]. Analysis was completed according to the six steps outlined by Braun and Clarke [46]. First, during the familiarization phase, two independent coders [NEE, SEM] immersed themselves in the data by reading through each interview twice. Second, initial codes were generated using an open coding technique to identify data extracts that were interesting or informative and which formed the basis of repeated patterns or themes. At the end of this phase, all codes within each theme were collated independently by each coder. Third, based on this open coding, the coders then met to identify “candidate” themes by developing a thematic map. Once candidate themes were identified, independent focused coding of each interview was completed, and data extracts were once again collated. Fourth, themes were then reviewed to ensure that they were both internally homogeneous (i.e., each theme was coherent) and externally heterogeneous (i.e., distinctions between themes were clear and identifiable). Fifth, each theme to be included in the final analysis was then named and defined. Lastly, the final analysis was written up based on the prompts identified by Braun and Clarke [46], including: What does this theme mean? What are its implications? What does the overall story of the different themes reveal about this topic?

### Ethics

Research ethics approval was provided by the Royal Ottawa’s Institute of Mental Health Research Ethics Board (IMHR-REB ID: 2017042) and the Ottawa Health Sciences Network REB (OHSN-REB ID: 20180895-01H).

## Results

### Participant flow

**Fig. 1:**
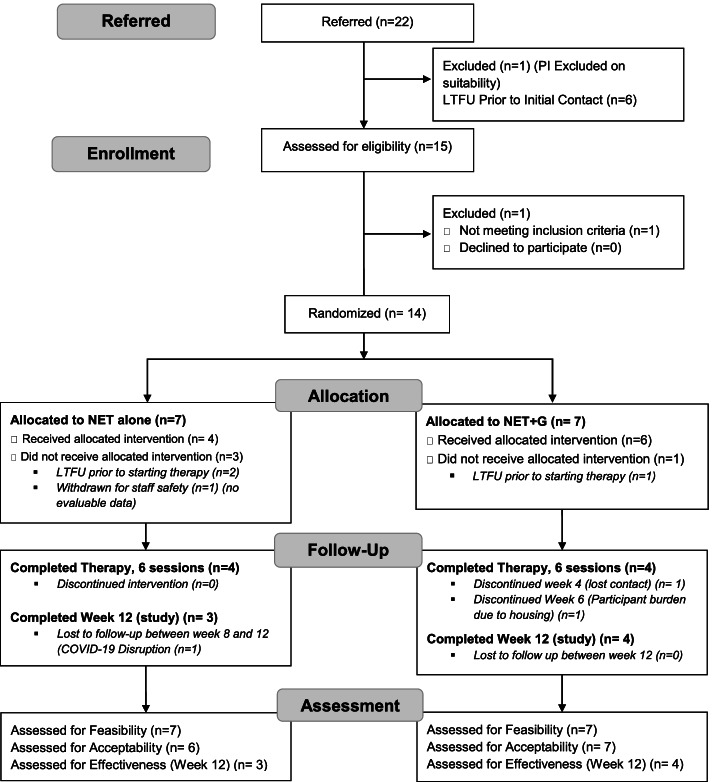
Consolidated Standards of Reporting Trials flow and attrition diagram

Participant flow is outlined in Fig. 1. Recruitment for this study took place between February 2019 and February 2020 and a total of 22 people were referred to the study (one of these was immediately before the first pandemic lockdown in late February 2020). Recruitment was stopped on two occasions, for a total of 3 months, as therapists had reached their maximum case load. Five participants were referred to the study after the start of the COVID-19 pandemic in March 2020, but we were unable to recruit them as there were no safe places to meet face to face as most drop-in centers or other spaces were closed because of COVID-19. We have not included them in the flow diagram. Virtual appointments were not an option for this population due to a lack of technology, access to the Internet, or safe and private places to conduct therapy.

The clinical teams referred 22 people to take part in the study over the 9 months that the study was accepting potential participants. One potential participant was deemed ineligible by the principal investigator due to a significant brain injury and associated psychosis. Six (6/22, 27%) potential participants could not be contacted to arrange a baseline visit and consent, leaving 15/22 (68%) who were assessed for eligibility for the study. One of these potential participants did not meet the inclusion criteria. This participant consented but was unable to complete the screening procedures. This led the team to evaluate the administration of screening measures to reduce burden on participants.

This left 14 participants who were randomized. Seven were randomized to NET and seven to NET+G. One participant who consented to the study and was randomized to the NET alone arm did not complete their baseline assessment. While consent, screening, and baseline typically occurred at one visit, this participant had a particularly long screening session, and the baseline assessments were scheduled to occur during a second encounter. The participant did not present for this visit and further information from the referrer showed a possible safety risk to the therapists, so this potential participant was withdrawn. This participant had no evaluable data and was not included in the evaluation of acceptability or effectiveness.

Three people were lost to follow-up between consenting to take part in the study and the first therapy session, one in the NET+G group and two in the NET group.

Every effort was made to provide participants with both their personal narrative and genealogy report before their final NET session. In particular, delays with genetic matching meant the first participant in the NET+G arm did not receive their report until after their final session. Adjustments were made in the timing of the genealogy interview so that all subsequent participants received their reports prior to completing therapy, and typically around week 4.

### Feasibility

We were unable to recruit our desired sample size within the goal of 6 months. The enrollment of 15 participants took place over approximately 9 months. This was primarily due to a shortage of trained therapists to take on participants. For the first 5 months, only one therapist was involved in the study. As the study progressed, we were able to recruit one additional trained therapist.

### Study acceptability

Figure 1 outlines the retention rates at various stages of the study. While we did not meet the a priori threshold of 75% retention at week 12, 61.5% (8/13) of participants completed at least one post-therapy follow-up assessment visit (week 8). Of the ten who started therapy, seven completed all study visits up to week 12. Of three participants who did not complete the week 12 assessment, the first completed the week 8 study visit and was interested in completing week 12; however, the final visit was rescheduled due to personal circumstances and was eventually canceled due to the COVID-19 pandemic; the second dropped out at week 6 as they found the sessions too triggering and difficult to complete while sleeping rough; and the third individual was lost to follow-up at week 4 but re-engaged with the clinical team at a later date.

### Therapy acceptability

Of the 10 participants who started therapy, eight (80%) completed all 6 sessions. All participants who were randomized to the NET+G arm (*n*=6) accepted the referral to complete their family history.

### Demographics

The demographics of participants are outlined in Table 4. Comparable to community demographics, our sample contained more males than females (61.5%, 8/13), with a lifetime history of drug use (84.6%, 11/13) and alcohol use (84.6%, 11/13). The sample self-identified primarily as white (65%, 8/13), but also First Nations (7.7%, 1/13), Métis (2/13, 15.4%), Asian (7.7%, 1/13), and other (Hispanic) (7.7%, 1/13). Within this identification, two people also identified themselves as mixed-ethnicity (Métis/White and First Nations/Black). Educational background was diverse with 38.5% (5/13) having less than high school, 30.8% (4/13) having a high school diploma or equivalent, 15.4% (2/13) with a college or university education, and 15.4% (2/13) with a graduate or professional degree. Marital status was also varied, with 46.2% (6/13) identifying as single, 7.7% (1/13) as married, 23.1% (3/13) as separated, 7.7% (1/13) as divorced, and 15.4% (2/13) as widowed. No individuals identified as transgender or non-binary. No participants indicated common-law status.

**Table 4:** Demographic characteristics

Demographic characteristic	Total (***n***=13) ***f*** (%)	NET (***n***=6) ***f*** (%)	NET+G (***n***=7) ***f*** (%)
**Gender**			
Male	8 (61.54)	3 (50.00)	5 (71.43)
Female	5 (38.46)	3 (50.00)	2 (28.57)
Age (years), mean (SD)	42.38 (8.15)	41.00 (4.34)	43.57 (10.66)
**Ethnicity**			
First Nations	1 (7.69)	0 (0)	1 (14.29)
Metis	2 (15.40)	1 (16.67)	1 (14.29)
Asian	1 (7.70)	1 (16.67)	0 (0.00)
White/Caucasian	8 (61.54)	3 (50.00)	5 (71.43)
Other	1 (7.7)	1 (16.67)	0 (0.00)
**Education**			
Below high school	5 (38.46)	2 (33.33)	3 (23.1)
High school or equivalent	4 (30.77)	1 (16.67)	3 (23.1)
College or university	2 (15.40)	1 (16.67)	1 (7.7)
Graduate or professional degree	2 (15.40)	2 (33.33)	0 (0.00)
**Marital status**			
Single	6 (46.15)	2 (33.33)	4 (57.14)
Married	1 (7.70)	0 (0.00)	1 (14.29)
Separated	3 (23.08)	2 (33.33)	1 (14.29)
Divorced	1 (7.7)	1 (16.67)	0 (0.00)
Widowed	2 (15.38)	1 (16.67)	1 (14.29)

### PTSD scores

All participants had experienced multiple traumatic events in their lifetime, with childhood trauma being common, as reported on the Life Events Checklist. With respect to symptom severity, baseline PCL-5 scores did not differ (*F*_(1, 13)_=1.169, *p* =.311) between those allocated to NET (*M* = 66.00, SD = 6.68) or NET+G (*M* = 60.67, SD=8.17).

PTSD scores decreased in both groups over the course of the study. Assessing the change in symptom severity within subjects for the 7 who completed post-therapy follow-up to week 12 showed a clinically meaningful change in PTSD scores (Table 5), defined as a reduction in total score by 10–20 points [47]. Prior to initiating therapy, the average PCL-5 score was 64.14 (SD 8.80). At the 12 week follow-up, participants reported an average decrease of 17.29 points (SD 16.63), for a total PCL-5 score of 46.86 (SD 16.63) (95% CI: 2.17–32.41).

**Table 5:** Within-subject outcomes

Time point	***PCL-5 scores*** *(n=7)*			***Drug use (previous 30 days)*** *(n=7)*			***Alcohol use (previous 30 days)*** *(n=7)*		
	***M***	***SD***	***Mean change***	***M***	***SD***	***Mean change***	M	SD	***Mean change***
Week 0	64.14	8.80	*M* = 17.29 SD = 16.35 95% CI: (2.17–32.41)	11.57	14.74	*M* = 0.86 SD = 2.27 95% CI: (−1.24–2.95)	1.00	1.73	*M* = 0.57 SD = 1.40 95% CI: (−0.72–1.86)
Week 12	46.86	16.63		10.71	14.27		0.43	0.787	

### Substance use

A lifetime history of substance use was common in this sample, with 84.6% (11/13) reporting a history of drug or alcohol misuse. At enrolment, 6 individuals reported current drug use (46.2%, 6/13) and 2 reported alcohol use (15.4%, 2/13) in the 30 days prior, while 5 participants reported no drug or alcohol use at all during this period (38.5%, 5/13). Alcohol or drug use did not change over the duration of the study (Table 5). Prior to initiating therapy, drug use was an average of 11.57 days (SD 14.74 days), while alcohol use averaged 1 day (SD 1.73 days). At week 12, drug and alcohol use was similar to baseline. Drug use had a mean change of nearly 1-day reduction (*M* = 0.86, SD = 2.27, 95% CI: [−1.24–2.95]) and alcohol use had a mean change of one half-day reduction (*M* = 0.57, SD = 1.40, 95% CI: [−0.72–1.86]).

Substance use appeared to have no relationship to whether or not a participant completed the study, with 23.1% of participants using drugs at enrolment completing the study (*p* = 1.00, Fisher’s exact test, 2-tailed) compared to 30.7% of non-users completing the study. Participants who did not complete the study were split, with 23.1% using drugs at enrolment and 23.1% not using drugs (Table 6).

**Table 6:** Study completion status

	Baseline ***N***=13	
	Study complete *f* (%)	Study not complete *f* (%)
Drug use (past 30 days)		
Yes	3/13 (23.1)	3/13 (23.1)
No	4/13 (30.7)	3/13 (23.1)
*Fisher’s exact test*	*p* = 1.00 (2-tailed)	
Alcohol use (past 30)		
Yes	2/13 (15.4)	0/13 (0)
No	5/13 (38.5)	6/13 (46.1)
*Fisher’s exact test*	*p* = .462 (2-tailed)	
Housing status		
Shelter/rough	2/13 (15.4)	5/13 (38.5)
Other	5/13 (38.5)	1/13 (7.6)
*Fisher’s exact test*	*p* = .103 (2-tailed)	

Similarly, no difference was found between those who completed the study and were using alcohol (2/13, 15.4%) or not using alcohol (5/13, 38.5%) compared to the six participants not using alcohol (6/13, 46.2%) who did not complete the study (*p* =.462, Fisher’s exact test, 2-tailed) (Table 6).

### Housing status

Housing status over the duration of the study is described in Table 7. At baseline, more than half of the participants were living in a shelter (53.8%, 7/13), followed by supportive/transitional housing (15.4%, 2/13), while 1 individual was living with a friend (7.7%), and another was paying for a living space (7.7%). Several individuals added specifiers to their situation including that they were paying for a living space because they received a subsidy or assistance from a family member. During the study, one individual (7.7%) left the shelter and was not able to find other housing options. Of those who did not complete the study, 38.5% (5/13) resided in a shelter baseline (*p* = .103, Fisher’s exact test, 2-tailed).

**Table 7:** Housing status

Housing status (*N* = 13)	Baseline	Week 4	Week 8	Week 12
	*f* (%)			
Shelter	7 (53.8)	3 (23.1)	4 (30.8)	3 (23.1)
With family	1 (7.7)	1 (7.7)	1 (7.7)	1 (7.7)
With friend	0 (0)	0 (0)	0 (0)	1 (7.7)
Supportive/transitional housing	4 (30.8)	2 (15.4)	1 (7.7)	1 (7.7)
Paying for a space	1 (7.7)	1 (7.7)	2 (15.4)	1 (7.7)
No housing options	0 (0)	1 (7.7)	0 (0)	0 (0)
Missing	0 (0)	1 (7.7)	0 (0)	0 (0)
Withdrawn	0 (0)	4 (30.8)	5 (38.5)	6 (46.2)

Of those who completed the study, 3 individuals remained at the shelter (3/7), while others were staying with friends (1/7), staying with family (1/7), living in supportive housing (1/7), or paying for a space (1/7). Several individuals experienced changes to their housing situation: two moved from supportive living to the shelter, one from the shelter to living with a friend, one from supportive housing to paying for a space, and one from living with family to paying for a living space. Of the 6 individuals who did not complete the study, 4 were at the shelter, 1 was “sleeping rough,” and 1 was paying for a living space.

### Qualitative evaluation

We were unable to complete qualitative interviews with the participants because of COVID-19 restrictions. There were no safe places to conduct interviews and using technology was not feasible as discussed above. Four service providers completed a virtual semi-structured qualitative interview with an unfamiliar research staff member. The service providers had varying backgrounds including two staff with Psychiatric Outreach who referred clients to the study, one manager at a shelter, and one manager at a community day program, both of whom facilitated study activities.

**Fig. 2:**
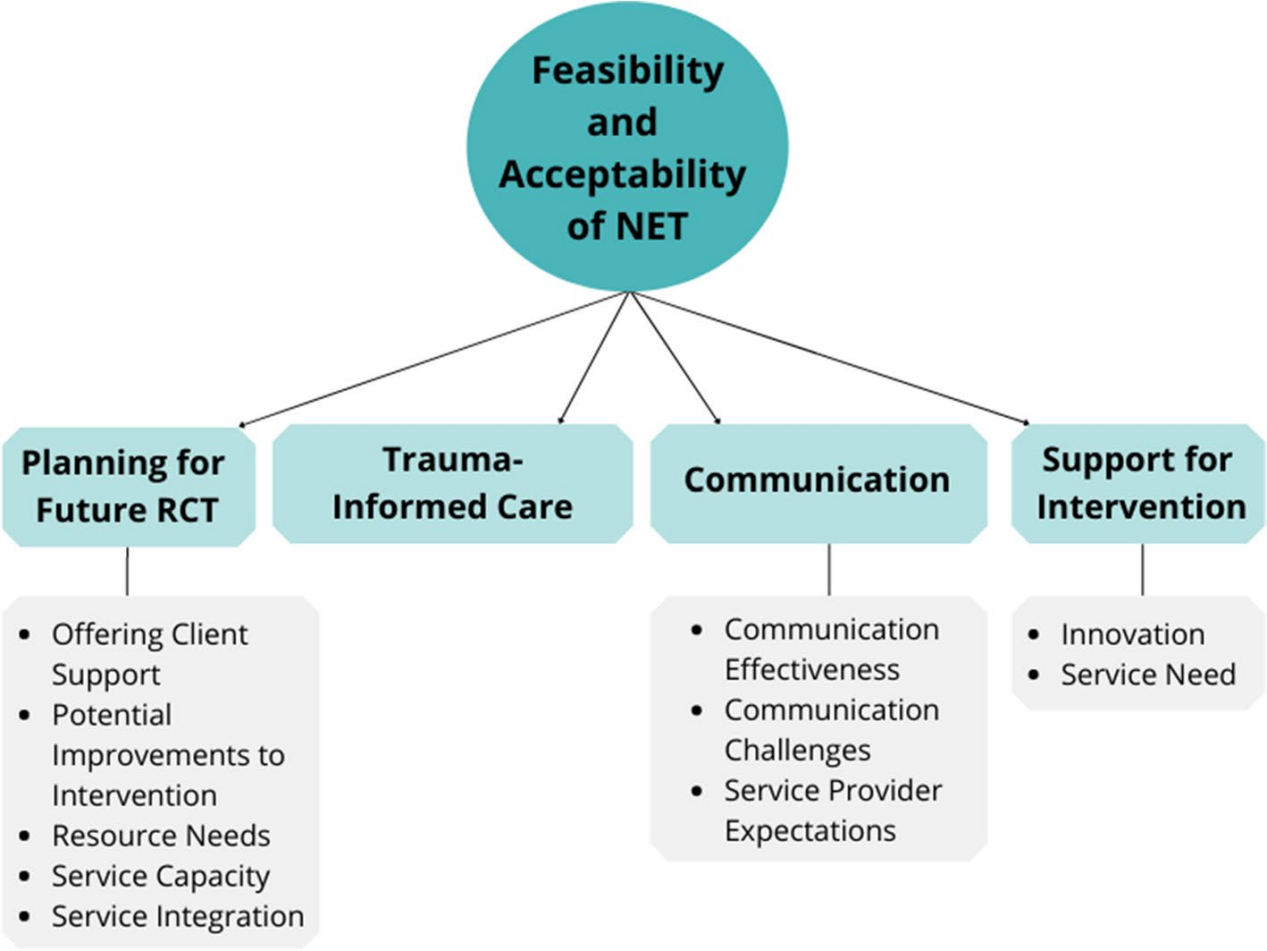
Thematic map illustrating theme and subtheme relationships

The patterns that emerged from the qualitative analysis have been organized under four broad themes: support for the intervention, planning for future RCT, communication and trauma-informed care, and several subthemes (Fig. 2). Detailed quotes for each theme can be found in Supplemental Table 1.

#### Support for intervention

Service providers all unanimously offered their support for the intervention. Many of the clients that these service providers work with have experienced trauma, but there is a distinct lack of treatment options available:

…It’s just more than needed, it’s irreplaceable, without it there’s nothing. Nothing like is really happening right now so it would be great to implement this in a community setting on a broader scale. Any additional help is needed so it’s great. [SP01 – outreach worker]

#### Trauma-informed care

All service providers interviewed highlighted components of trauma-informed care, including trauma awareness, safety, and choice, that they felt supported the client experience during this study. Interviewees highlighted the importance of understanding the prevalence and impacts of trauma and also discussed how to best ensure the physical and emotional safety of their clients:

Also speaking to the community setting piece, definitely there should be more services at our location or shelters in general because a hospital setting isn’t always welcoming or comfortable and many actually have bad experiences there so this setting makes them more willing to partake so definitely yes to the community setting part [SP04 – shelter residential services manager]

#### Communication

Study communication was primarily through email for referrals, organizing visits, and follow-up conversations. Service providers described communication with the team as “prompt,” “friendly,” and feeling that they were “never left hanging.” While service providers all indicated that email would be their preferred method of communication, they each noted that this method of communication would pose challenges with their clients. Using a flexible, solutions-focused approach to communication, the team often collaborated with service providers, offering a trusted point of contact, to address these challenges with clients. A frontline service provider described such an instance:

Of course there are sometimes issues with this population and contacting them by email because they don’t always have access and I can remember in one particular case I can remember a client was homeless and didn’t manage well in shelters and chose to live in a tent and use a drop in centre, so for that particular person, connecting via email was tricky to find the client but we worked together with the client to coordinate that and it was fine in the end. [SP02 – outreach worker]

Expectations of being involved in the study were largely understood by service providers and those expectations were met throughout the study. One service provider did note that their expectations were not very clear at the outset, but they remained open-minded throughout the study. Communication of expectations throughout the duration of any future studies will be important for success.

…I guess my expectations weren’t actually very clear, I was just excited for this new place that we could refer clients and hopefully open to the door to some type of care we currently weren’t offering. [SP02 – outreach worker]

#### Planning for a future RCT

Planning for the optimization phase of the MOST strategy was a key outcome of this pilot study. Ensuring that their clients remain supported was of significant importance to the service providers. In discussing the study and the intervention, service providers noted that the intervention served their client’s best interests and highlighted their relentless efforts to ensure their client received the best care possible:

…there is such a gap in services for clients so as an outreach worker when we’re meeting with people and they’re looking for support let’s say in particular for their PTSD to try and find treatment, there’s a lack of services to refer them to and you leave no stone unturned when you’re faced with a story and situation to try and connect a person to services [SP02 – outreach worker]

Service providers described areas for improvement with the intervention, primarily relating to the study procedures and not the therapy itself. Outreach workers noted wanting increased engagement throughout the study, with one interviewee highlighting that this would better support clients and also had the potential to increase engagement by acting as a trusted source:

See once the client was referred to the study, it didn’t mean they were still engaged with me or any outreach worker, so maybe it would be beneficial if the outreach worker was more involved in the entire process and depending that might yield a bigger turn out for clients coming to their appointments but again once referred I’m not sure what happened after that so that could be a gap that might be addressed in that way. [SP01 – outreach worker]

Service providers also spoke to the need for clarity around how the study would impact their organization’s internal processes. Service providers described low service capacity, understood as the ability for existing services to offer the intervention, note a shortage of staff with appropriate qualifications to deliver therapy or the bandwidth to offer a new treatment. However, service integration, defined here as the ability for existing services to combine with external resources to offer the intervention, was met with more encouragement. Most service providers indicated that NET and the research study fit in well within their existing programs. Interviewees highlighted the importance of providing clients with a range of possible supports:

I don’t know our staff are trained or qualified to provide therapy even with training, but we’d do what we can and definitely would add and support in any way we could yeah I think that’d be great [SP04 – shelter residential services manager]

To be able to successfully implement a large RCT, various types of resources would be required, including financial support, physical space, and personnel commitments. Service providers highlighted a variety of resources they considered critical to move forward with a new study.

I think that would be a great idea but in terms of positions and funding, you never know but we’d always love more support so it would be great, like my co workers and I would be gung ho about that but management might have a different opinion about that so I’m not too sure. [SP02 – outreach worker]

While the current study was well-received by those that participated, several actionable recommendations will be incorporated during the optimization phase.

## Discussion

### Generalizability

The sample enrolled in the study is largely representative of the homeless population, both in Ottawa and nationally. However, given the small sample size, it would be unwise to extrapolate the results of this study to widely. What it does show is that it is possible to recruit a representative sample into a RCT.

### Interpretation

Individuals experiencing homelessness have long been excluded from clinical trials with the assumption that they would be challenging to engage or retain. We found that, while not quite meeting the pre-defined thresholds for feasibility and acceptability, conducting an RCT of a mental health intervention in the community was possible. To improve feasibility and acceptability during a large-scale RCT, several lessons learned from this pilot, including therapist capacity and the importance of a trauma-informed care approach, can be used to improve both engagement and retention.

A lack of therapists trained in NET had the largest impact on meeting our feasibility target of recruiting 24 individuals within 6 months. The COVID-19 pandemic also impacted recruitment with no individuals enrolled after February 2020. NET training is limited with only 2 offerings occurring in the Ottawa area within the 3-year study period. The principal investigator [SH] recently organized two virtual training sessions with high levels of community interest. Building therapist capacity will be critical to the success of implementing a large RCT and in offering NET as a standard of care long term in the community. The provision of NET by health professional students supervised by experienced therapists is also a model that needs to be explored, especially given most universities’ commitment to social accountability in their communities.

While our initial target of 75% retention was not met, our retention rate is consistent with literature around engagement in psychotherapy, while our proportion of participants for completing all sessions of therapy (80%) exceeded average retention rates [48, 49]. Given the considerations of working with individuals who are homeless and the potential challenges of engaging with trauma therapy, we feel that these rates are high enough to deem the therapy and participation in a study to be acceptable.

What is striking is that the drop-out rate before starting therapy is much higher than after starting treatment, with 12/22 (55%) of potential participants dropping out prior to therapy compared to only two out of ten (20%) after starting therapy. It is likely that this is due to a combination of three factors. First is that referrers to the study need to be given clear guidance as to who is potentially eligible and who is not. Also providing information about what happens after therapy is completed would be helpful. Second, there are real practical difficulties in contacting people who are vulnerably housed in a way and at a time which is convenient to them. Third, there are the issues of credibility and trust. People who have experienced trauma, especially in childhood, have learnt not to trust carers and many may have had bad experiences in their contacts with the health system. The contractual process of signing an informed consent form is probably not sufficient to gain trust without other actions. These could include paying attention to the privacy of data, linking the study to credible organizations, and being clear about who has what role in the research team and how long participation will last. Requiring potential participants to see several unfamiliar people before starting therapy may also impact on trust. Training research assistants in helping people with complex PTSD and its impact on relationships is important. This would be guided by trauma-informed research practices, such as work done by Voith and colleagues [50], and would seem to be an important component of a bigger trial.

Substance use at the time of enrolment did not appear to impact whether or not a participant was likely to complete therapy and follow-up sessions, suggesting that it is feasible to include those using substances in research and therapy. However, staying in a shelter at the time of enrolment did show some potential impact with respect to not completing the study. Given this, consideration should be given to how to best support therapy retention when working with individuals who are staying in a shelter. Factors to increase retention should be examined during the optimization phase of MOST, with a particular focus on those who are in the shelter or sleeping rough.

Within-subject differences showed a clinically meaningful reduction in PTSD symptoms in participants from baseline to week 12, suggesting that NET may be effective in this population. Additionally, while the impact of the genealogy reports was not able to be assessed due to the small sample size and the inability to complete qualitative interviews, no participants refused to complete the interview. One participant who had dropped out at week 4 re-approached the study team several weeks later hoping to obtain their report. Further research through a large-scale RCT is needed to determine the effect of NET augmented by genealogy and optimize the delivery of NET in this population.

### Strengths and limitations

The major strength of this study is that we have shown that it is possible to conduct an RCT for the treatment of trauma with individuals experiencing homelessness in a community setting — the first study of its kind. The decrease in PTSD scores suggests that NET is an effective treatment in this population, as it is in people who are not vulnerably housed. The major limitation of this study was a lack of individuals trained in NET which resulted in potential participants not being enrolled in a timely way. As a pilot feasibility study, the trial was underpowered to detect any significant differences between treatment groups. Also, we did not distinguish between PTSD and complex PTSD in the inclusion criteria and the possible different effects the treatment may have on these two subtypes of PTSD. In practice, all participants in the study had complex PTSD.

With respect to the genealogy report, it took approximately 4–6 weeks to receive a completed report, which made it challenging to incorporate the findings of the report into the narrative process. It would be possible in a large-scale trial to ensure that the individual is seen by the genealogist in advance of initiating therapy to increase the likelihood of receiving the report during active narration.

Lastly, due to the sudden lockdown measures implemented during the COVID-19 crisis, the study team was unable to complete the planned qualitative interviews about participant experiences in the study, the therapy, or genealogy support. Anecdotally, there were some complaints about the complexity of two questionnaires (SF-20 and Health Care Costs Questionnaire), but participants did not refuse to answer any questionnaires or comment on the length of visits. With respect to the genealogist, participants all received their reports, with one participant lost to follow-up requesting their report several months later. Only one participant, at the time of consent, indicated that they would have no interest in speaking with a genealogist if they were randomized to that arm.

### Clinical considerations

There is currently a significant gap in trauma treatment for the homeless community, despite the high prevalence and degree of complexity within this population. This study has shown that not only is it feasible to deliver community-based therapy without a fixed location, but that Narrative Exposure Therapy is potentially an effective and acceptable therapeutic option for individuals experiencing complex trauma and homelessness. While qualitative results are not available to support acceptability due to the COVID-19 pandemic, the low drop-out rate observed after participants began therapy suggests that they consider the therapy acceptable.

Service providers also emphatically endorsed the need for this program after their participation in the study. Interviews completed at the end of the study highlight the current lack of services, support for broad implementation, the importance of a trauma-informed approach, and recommendations to improve future iterations of this project.

## Conclusions and future research

This study was the first of three components of the preparation stage of a multiphase optimization strategy (MOST). The second is a scoping review [41] which we have published separately on the treatment of PTSD in the homeless including the use of trauma-informed care to deliver such therapy. This scoping review found no RCTs of trauma-focused psychotherapies (like NET), an overall lack of high-quality trials to address PTSD in this population, and elements of safety, the experience of being heard, and flexibility of choice as being important components of trauma-informed care when providing treatment. The third is a consultation with key stakeholders, including individuals with lived experience, about the implementation of a trauma-informed care model for individuals who are homeless or vulnerably housed. A focus of this consultation has been addressing the issue of trust when referring potential participants to a treatment study. We will use these components to develop a model of delivering NET in this population which we will optimize using a multi-site factorial RCT. A key component of the MOST strategy as described by Collins [39], the factorial RCT, will evaluate multiple intervention combinations, including elements of trauma-informed care and other factors as identified during the preparatory phase. This factorial analysis will result in an optimized intervention which can then be tested in a definitive trial of vulnerably housed people with complex PTSD comparing NET alone versus NET augmented by a genealogical assessment. The multi-site RCT will also help to evaluate issues of generalizability across communities that have different supports for their homeless population.

## Supplementary Information

**Additional file 1.** Coded quotes from service provider interviews.

## Data Availability

The datasets used and/or analyzed during the current study are available from the corresponding author on reasonable request.

## Abbreviations

NET: Narrative Exposure Therapy
RCT: Randomized controlled trial
PTSD: Post-traumatic stress disorder
cPTSD: Complex post-traumatic stress disorder
MOST: Multi-phase optimization strategy
NET+G: Narrative Exposure Therapy plus genealogy
OHRI: Ottawa Hospital Research Institute
