# Supplementary material for: Feasibility and acceptability of Narrative Exposure Therapy to treat individuals with PTSD who are homeless or vulnerably housed: a pilot randomized controlled trial
Source: Pilot Feasibility Stud. 2022 Apr 15;8:83. doi: 10.1186/s40814-022-01043-x (PMC9010708; doi:10.1186/s40814-022-01043-x)
Supplement: Supplementary file 1 — Additional file 1. Coded quotes from service provider interviews. [file 40814_2022_1043_MOESM1_ESM.docx]

**Supplemental Table 1. Coded quotes from service provider interviews.**

| **Theme -Subtheme** | **Number of References** | **Participant ID - Quote** |
| --- | --- | --- |
| Communication - *Communication Challenges* | 7 | SP01- Do you mind expanding on the barriers involved with email communication for the clients in terms of email communication?  P: They didn’t have access to the technology like internet or supporting devices as is in many cases with his population it can be difficult to communicate generally. |
|  |  | SP01 - I don’t know if clients were following up always |
|  |  | SP01 - Challenges would be contacting and keeping in touch probably with this population but again personally, I didn’t have any challenges. |
|  |  | SP02 - Of course there are sometimes issues with this population and contacting them by email because they don’t always have access and I can remember in one particular case I can remember a client was homeless and didn’t manage well in shelters and chose to live in a tent and use a drop in centre, so for that particular person, connecting via email was tricky to find the client but we worked together with the client to coordinate that and it was fine in the end. |
|  |  | SP02 - Any communication with this population can sometimes be tricky |
|  |  | SP02 - What could be done differently to reduce or eliminate those challenges so the administrative piece and the referring?  P: Just some clarity outlined by the research staff would be really helpful but overall it was great and great to work with them and this was so exciting and I’d love to see it continue. |
|  |  | SP03 - …but it can be challenging for folks visiting the Centre, I wouldn’t say it’s the method though that causes an issue but just generally if someone doesn’t have a phone or an address where we can contact them it can be a challenge but… |
| Communication - *Communication Effectiveness* | 6 | SP01 - Okay, and what are your perceptions of communication with the study team so Dr. Hatcher for example the Principal Investigator or other study staff members?  P: It was logistical, if I just keep it isolated to the study, it was just to get involved with the study, get the client involved with the study, to proceed with that narrative counselling. But it was good, I had no issues with it.  I: Okay and what was the most frequent method or methods of communication when interacting with the study team?  P: Email  I: Okay, and are there challenges or limitations with these forms of communication, so in this case, email?  P: For my clients there would be, but for myself no. |
|  |  | SP02 - Moving into the next question here, what were your perceptions of communicating with members of the study team?  P: Yeah so the two main people I communicated with would be Nicole for the referrals and processes, and Simon. So I found the communication was perfect and I had absolutely no issues there, they always responded in a timely manner and I was never left hanging so no feedback there at all, it was great.  I: Perfect and what was the most frequent methods of communication so phone, email, in person?  P: Generally email, sending referrals and communicating with Nicole about what they need or are looking for, questions about PTSD questionnaires, anything really was over email.  I: Okay great and would you say there are challenges or limitations with these methods of communication?  P: For me, no email works best and is and was a great way to communicate with the study team |
|  |  | SP02 - but in terms of emailing with us as a staff, no it’s a preferred way to communicate |
|  |  | SP03 - The study team was good at communicating and when they needed to were able to use our Centre so I’d say my expectations were met for sure.  I: Okay awesome, thank you. Moving on to the next question, what were your perceptions of communicating with members of the study team?  P: Communication with the team was great, I was in contact mainly with Nicole and she was always pleasant to work with. She’s prompt at answering emails, she is friendly and does everything on her end that needs to be done so it was good, no issues with communication at all.  I: Great and what was the most frequent methods of communication as in phone, email, in person?  P: Email was pretty much always how we communicated, it’s been a while I forgot maybe by phone occasionally but there was never a time I needed to contact her or Simon and couldn’t. I also saw Nicole when she was in at the Centre and she was always good and communicating and super friendly. Email generally works best if I’m doing something else I can get to it when I return to my desk sort of thing so it was good or she could tell me info when she was in if there was something she needed or wanted to update me about.  I: Okay great and would you say there are challenges or limitations with these methods of communication?  P: I didn’t have any challenges communicating with email or even phone/in person, |
|  |  | SP03 - I think we make it work and communicating with Nicole and Simon wasn’t challenging or limiting. |
|  |  | SP04 - What were your perceptions of communication with the study team?  P: Good, very good, no issues at all, I’d talked with both Nicole and Simon and I never had issues with communication  I: Okay fantastic and what would you say was the most frequent methods of communication, as in email, phone, in person, or any other way you may have communicated?  P: By email for sure, really the only way I communicated about this study  I: Okay great and are there any challenges or limitations with these methods of communication?  P: I don’t think there were any issues, on my end anyways I never had issues, not sure for them if I didn’t answer quick enough or anything but for me, no issues or limitations |
| Communication - *Service Provider Expectations* | 9 | SP01 - what were your expectations as an outreach worker?  P: I guess my expectation was that if the client was chosen for the research that they would have an opportunity to start to engage with some counselling and start to enter in to that relationship with professionals in order to deal with past trauma and homelessness and I would help facilitate that. |
|  |  | SP01 - did your involvement meet these expectations?  P: I believe yes, a couple clients went to the study and experienced what I had anticipated so I would say my expectations were met. |
|  |  | SP02 - my expectation was simply that there would be a new resource to refer clients to that could potentially help them and it was a resource we didn’t have before so it was worth a try. |
|  |  | SP02 - I guess my expectations weren’t actually very clear, I was just excited for this new place that we could refer clients and hopefully open to the door to some type of care we currently weren’t offering |
|  |  | SP02 - we were referring everybody so my question would be like who’s a great candidate so I know I’m straying a little bit but since I had unclear expectations I was open minded and excited so I’d say I didn’t quite have expectations to meet but was happy this happened |
|  |  | SP02 - to summarize yes it met my expectations but I expected a little bit more guidance in terms of who exactly should be referred. |
|  |  | SP03 - So I expected to help provide a place for the study team to meet and do their therapy and study activities here at the Centre so they’d have a place that was somewhat familiar and safe to connect in  I: Okay great, any other expectations pertaining to the study?  P: No that was really it  I: Okay no problem, did your involvement meet these expectations?  P: Yes, they did, this was a meeting point and I had no issues or anything like that. The study team was good at communicating and when they needed to were able to use our Centre so I’d say my expectations were met for sure. |
|  |  | SP04 - So, what were your expectations of being involved in the NET study?  P: To be honest I was just asked if I could provide referrals for Dr. Hatcher and that’s really it |
|  |  | SP04 - did your involvement meet those expectations?  P: Yeah, that’s exactly what I did, no surprises, I just referred when I thought it was a fit and that was kind of my only role there |
| Planning for Future RCT - *Offering Client Support* | 5 | SP01 - there would have to be someone to liaison the client with the study to be able to access and know about it |
|  |  | SP01 - what about allowing for a centralized team member to come into your location to provide therapy services?  P: Again, I think it serves the clients best interest so I don’t see that being turned down by anyone, |
|  |  | SP02 - there is such a gap in services for clients so as an outreach worker when we’re meeting with people and they’re looking for support let’s say in particular for their PTSD to try and find treatment, there’s a lack of services to refer them to and you leave no stone unturned when you’re faced with a story and situation to try and connect a person to services |
|  |  | SP02 - When I heard about the Net study it was just really exciting because there was a place where this person could start care and actually have someone to talk to but their maybe new or long lasting diagnosis. |
|  |  | SP04 - I’m all for it, really any work we can do in progressing clients in any way, I’m open to, so this is just another good resource to try and help people and of course the vulnerability here, I think it’s great. |
| Planning for Future RCT - *Potential Improvements to Intervention* | 6 | SP01 - but that was a piece that I think would be important is finding the right people to help liaise the entire process to keep clients engaged. |
|  |  | SP01 - See once the client was referred to the study, it didn’t mean they were still engaged with me or any outreach worker, so maybe it would be beneficial if the outreach worker was more involved in the entire process and depending that might yield a bigger turn out for clients coming to their appointments but again once referred I’m not sure what happened after that so that could be a gap that might be addressed in that way. There might be better compliance but again I don’t know if the outreach worker is supported to deal with that type of therapy or involvement. |
|  |  | SP02 - we were referring everybody so my question would be like who’s a great candidate so I know I’m straying a little bit but since I had unclear expectations I was open minded and excited so I’d say I didn’t quite have expectations to meet but was happy this happened and if it were to move forward maybe we could have some more clear guidance because for narrative therapy in particular some were a fit and some weren’t so I was kind of referring while hoping I was doing the right thing. |
|  |  | SP02 - I’m happy that we did that because I think it opens the door to more questions and that can improve the study hopefully about some guidelines about who exactly fits within it. For example if you had someone with PTSD and a brain injury or PTSD and something else, should they still be referred, like is that appropriate |
|  |  | SP02 - so the only one thing that was in question, was the length of time, so our service is only suppose to keep files open for three months at max right so the question becomes do we just do the referral and close the file or do we keep it open. So this particular process would need refining and some guidance and something that could be further assessed so to assure the client has appropriate coverage. |
|  |  | SP02 - I think just in general is making sure that we’re referring the right person and it was sometimes hard to know if it’s a complex case or client. Then just administrative piece, do we keep the case open or closed that kind of thing like I mentioned before but other than that not really. |
| Planning for Future RCT - *Resources Needed* | 13 | SP01 - there would also need to be an environment and physical space for this type of thing to occur that is accessible |
|  |  | SP01 - outreach workers would definitely have to be involved to keep it going and play a facilitating role |
|  |  | SP01 - I don’t know if clients were following up always but that was a piece that I think would be important is finding the right people to help liaise the entire process to keep clients engaged. |
|  |  | SP01 - I don’t know if clients were following up always but that was a piece that I think would be important is finding the right people to help liaise the entire process to keep clients engaged. |
|  |  | SP01 - I don’t see there being any negative reaction to proposing that but whether the right people are willing to organize that and arrange funding and resources is a complex question that I can’t really say a definite yes or no. |
|  |  | SP02 - the challenge isn’t figuring out if there’s a need but more how can we provide what’s needed so definitely a need. |
|  |  | SP02 - if this were to be implemented on a larger scale, who are the key individuals to get on board, either at your location so the Royal or within the broader community, with delivering psychotherapy in the shelter or community settings?  P: Well probably the psych outreach team at the Royal, inner city, shelters, I’d say CMHA, they do a lot of great work, probably not everyone but you could find contacts there and they also leave no stone unturned. So from I was think of and provide knowledge about like the people referring, running the therapy, the shelters and then even clients maybe get their feedback about it, even though that can be hard I know. |
|  |  | SP02 - Probably a source of funding as well but I’m not really sure where that would come from, sorry. |
|  |  | SP02 - would you expect to have sufficient resources to implement and administer this type of therapy with your own staff at the royal, if training was provided?  P: Well I think it would be exciting, would management go for that in terms of providing time and funding and all that logistical stuff, I can’t say, I’d like to hope so but that’s kind of out of my hands. But I would love it and think it would be beneficial if we could. |
|  |  | SP02 - what about allowing a centralized team member to come into your location to provide the therapy services.  P: Again, I think that would be a great idea but in terms of positions and funding, you never know but we’d always love more support so it would be great, like my co workers and I would be gung ho about that but management might have a different opinion about that so I’m not too sure. |
|  |  | SP03 - if this were to be implemented on a larger scale, who are the key individuals to get on board, either at your location so Centre 454 or within the broader community, with delivering psychotherapy in the shelter or community settings?  P: Um, well in terms of community we would be happy to discuss further about navigating, it’s hard right now to do anything in person with COVID but you’d probably want to speak to more health professionals so I’m not sure I have a lot to offer on this question, sorry. But I would be happy to discussing and provide a physical space generally but yeah. |
|  |  | SP03 - would you expect to have sufficient resources to implement and administer this type of therapy with your own staff, if training was provided?  P: Overall, I think so. I mean we have the space so it would be more time as a resource to look at but I’m sure we could navigate that and work it out for sure but we would need the actual therapy staff right |
|  |  | SP03 - what about allowing a centralized team member to come into your location so Centre 454, to provide the therapy services?  P: Yeah I think that would be great idea, I’m not sure about funding for us but if someone were to be sent in, I don’t see that being an issue. |
| Planning for Future RCT - *Service Capacity* | 4 | SP01 - Would you expect to have sufficient resources to implement and administer this type of therapy with your own staff, if training was provided?  P: There might be, there might be initiative enough, its innovative, it’s out there in the street I’d think that there could be some momentum to get behind it. |
|  |  | SP02 - would you expect to have sufficient resources to implement and administer this type of therapy with your own staff at the royal, if training was provided?  P: Well I think it would be exciting, would management go for that in terms of providing time and funding and all that logistical stuff, I can’t say, I’d like to hope so but that’s kind of out of my hands. But I would love it and think it would be beneficial if we could. |
|  |  | SP03 - would you expect to have sufficient resources to implement and administer this type of therapy with your own staff, if training was provided?  P: Overall, I think so. I mean we have the space so it would be more time as a resource to look at but I’m sure we could navigate that and work it out for sure but we would need the actual therapy staff right |
|  |  | SP04 - I don’t know our staff are trained or qualified to provide therapy even with training, but we’d do what we can and definitely would add and support in any way we could yeah I think that’d be great. |
| Planning for Future RCT - S*ervice Integration* | 9 | SP01 - how well dos the Net study fit with existing work processes and practices in your setting?  P: Excellent, it gives us another option for clients to get follow up and another point of care that can benefit them and help them deal with their trauma. |
|  |  | SP01 - what about allowing for a centralized team member to come into your location to provide therapy services?  P: Again, I think it serves the clients best interest so I don’t see that being turned down by anyone, |
|  |  | SP01 - there are a variety of providers at the Royal if there was a service missing and another provider was able to offer something extra I think that would be great. |
|  |  | SP02 - But the project definitely aligns with what we’re doing for sure. |
|  |  | SP02 - what about allowing a centralized team member to come into your location to provide the therapy services.  P: Again, I think that would be a great idea but in terms of positions and funding, you never know but we’d always love more support so it would be great |
|  |  | SP03 - I think it was great, I mean in terms of coordinating we could do it again if the study were to run again and I think it’s a great service to offer to this population and we have the type of set up for it, |
|  |  | SP03 - allowing a centralized team member to come into your location so Centre 454, to provide the therapy services?  P: Yeah I think that would be great idea, I’m not sure about funding for us but if someone were to be sent in, I don’t see that being an issue. |
|  |  | SP04 - I’m not involved in the study or care so I’m not sure beyond my role in referring but I’d continue to refer for sure and the clients here would definitely benefit so I think the process at least works for me. |
|  |  | SP04 - we would love to have a team member come in and provide these types of services for sure and we could make time for staff meetings and organize this type of thing, I think that would be great. |
| Support for Intervention | 15 | SP01 - There is no negative to trying this as an avenue |
|  |  | SP01 - This also offered another option to redirect with trauma where the outreach worker isn’t really trained for it so again not a barrier or challenge, just a good option for clients. |
|  |  | SP01 - it was just nice to have this as an option to offer the client you know there’s a lot of people with trauma out there and they want to deal with it but they need and want professional guidance so this was great |
|  |  | SP02 - my expectation was simply that there would be a new resource to refer clients to that could potentially help them and it was a resource we didn’t have before so it was worth a try. |
|  |  | SP02 - When I heard about the Net study it was just really exciting because there was a place where this person could start care and actually have someone to talk to but their maybe new or long lasting diagnosis. |
|  |  | SP02 - would you expect to have sufficient resources to implement and administer this type of therapy with your own staff at the royal, if training was provided?  P: Well I think it would be exciting, would management go for that in terms of providing time and funding and all that logistical stuff, I can’t say, I’d like to hope so but that’s kind of out of my hands. But I would love it and think it would be beneficial if we could. |
|  |  | SP02 - like my co workers and I would be gung ho about that |
|  |  | SP02 - Just some clarity outlined by the research staff would be really helpful but overall it was great and great to work with them and this was so exciting and I’d love to see it continue.  I: Lastly, please feel free to provide any additional comments, thoughts, criticism, suggestions that we maybe didn’t chat about?  P: No I think we covered it and it was so nice to be a part of I hope this can be implemented on a larger scale and the team was great |
|  |  | SP03 - I think it’s a great service to offer to this population and we have the type of set up for it, |
|  |  | SP03 - Last one, please feel free to provide any additional comments, thoughts, criticism, suggestions that we maybe didn’t chat about?  P: No I think we covered it, everyone was lovely to work with and I like the idea behind this project so I’d be interested to see if you carry it out further and to more people, but overall I had a great experience. |
|  |  | SP04 - Good I guess, because we have Dr. Hatcher in there for other things so he’s wearing different hats sometimes when he’s in there for different things but any time I can get him or his ideas in the building I’m all for it, really any work we can do in progressing clients in any way, I’m open to, so this is just another good resource to try and help people |
|  |  | SP04 - Also speaking to the community setting piece, definitely there should be more services at our location or shelters in general because a hospital setting isn’t always welcoming or comfortable and many actually have bad experiences there so this setting makes them more willing to partake so definitely yes to the community setting part |
|  |  | SP04 - Yeah for sure COVID has changed many things and we’re in different times, what about if COVID were not a factor though, any feedback there?  P: To be honest, I don’t know who you’d need to contact, we’d be on board to do our part in referring and things like that but I think that question would be directed to more so the people providing care and individuals like that |
|  |  | SP04 - Would you expect to have sufficient resources to Implement and administer this type of therapy with your own staff, if training was provided or allow for a centralized team member to come into your location to provide the therapy services?  I: Oh for sure yeah, we would love to have a team member come in and provide these types of services for sure and we could make time for staff meetings and organize this type of thing, I think that would be great. I don’t know our staff are trained or qualified to provide therapy even with training, but we’d do what we can and definitely would add and support in any way we could yeah I think that’d be great. |
|  |  | SP04 - Lastly, please feel free to provide any additional comments, thoughts criticisms or suggestions.  I: That’s really it, we’ve covered it, I am happy I could be a part of this, Simon is always great to work with and yeah that’s really it I don’t have any comments or anything. |
| Support for Intervention - *Innovation* | 3 | SP01 - Nothing like is really happening right now so it would be great to implement this in a community setting on a broader scale. |
|  |  | SP01 - Would you expect to have sufficient resources to implement and administer this type of therapy with your own staff, if training was provided?  P: There might be, there might be initiative enough, its innovative, it’s out there in the street I’d think that there could be some momentum to get behind it. |
|  |  | SP02 - You leave no stone unturned right it’s like I’ll do whatever I can to help them so you want to use all resources, especially new exciting ones that could possibly be a fit. |
| Support for Intervention - *Service Need* | 3 | SP01 - how would you describe the need for this type of service, so therapy delivered in a community setting, specifically Narrative Exposure Therapy for individuals experiencing PTSD?  P: It’s just more than needed, it’s irreplaceable, without it there’s nothing. Nothing like is really happening right now so it would be great to implement this in a community setting on a broader scale. Any additional help is needed so it’s great. |
|  |  | SP02 - how would you describe the need for this type of service, so more specifically Narrative Exposure Therapy in a community setting for individuals with PTSD?  P: Absolutely necessary, nothing to really add, there needs to be this type of service for this population |
|  |  | SP04 - because I’m not a healthcare professional but I guess what I can say is that the individuals who struggle with mental illness need any resources or help they can get so I think we need more services like this generally |
| Trauma Informed Care | 5 | SP01 - Well dealing with the trauma aspect makes clients susceptible to a level of discomfort and dealing with those issues delicately |
|  |  | SP02 - Of course there are sometimes issues with this population and contacting them by email because they don’t always have access and I can remember in one particular case I can remember a client was homeless and didn’t manage well in shelters and chose to live in a tent and use a drop in centre, so for that particular person, connecting via email was tricky to find the client but we worked together with the client to coordinate that and it was fine in the end. |
|  |  | SP03 - I think it’s a great service to offer to this population and we have the type of set up for it, especially because some people don’t want to go in to a Hospital the community setting is more comfortable so I think it works well.  I: Okay great, and how would you describe the need for this type of service, so more specifically Narrative Exposure Therapy in a community setting for individuals with PTSD?  P: I think it’s great, again sometimes this population doesn’t have the opportunity or they don’t feel comfortable in a healthcare setting so I think it would be great |
|  |  | SP03 - Do you think there were there any barriers or challenges to being involved in the NET Study?  P: Um, I didn’t have any barriers or challenges really, no. I think involvement might be one because sometimes it’s hard to get a commitment but in my involvement I didn’t face any challenges.  I: Okay, and what could be done differently to reduce or eliminate those challenges so the commitment, if you have any suggestions?  P: I don’t know if there’s much we can do because it’s how their feeling whether they feel comfortable coming in right we can’t force it but just making our space comfortable and open for these types of things I think is good. |
|  |  | SP04 - Also speaking to the community setting piece, definitely there should be more services at our location or shelters in general because a hospital setting isn’t always welcoming or comfortable and many actually have bad experiences there so this setting makes them more willing to partake so definitely yes to the community setting part |
